# On the Alkaline Method of Treatment in Acute Rheumatism

**Published:** 1856-10

**Authors:** James Graham

**Affiliations:** Professor of Materia Medica and Therapeutics, in the Medical College of Ohio


### SELECTIONS.

*On the Alkaline Method of Treatment in Acute Rheumatism.* By JAMES GRAHAM, M.D., Prof. of Materia Medica and Therapeutics, in the Medical College of Ohio.

Dr. John H. Griscom, one of the Physicians to the New York Hospital, has recently presented to the profession the results of his experience in the treatment of acute rheumatism by the alkaline method, and these results correspond so closely with my own at the City Infirmary, during my late connection with that Institution, that I venture to offer a few general remarks on the subject.

Dr. Griscom's statistics comprise twenty-six cases, and the average time that his patients were under treatment was about thirteen days; the duration of the whole attack averaging about twenty-one days. Dr. G. asserts, that under the most improved plans of treatment heretofore pursued, the average duration of treatment has been about six weeks. There is one point to which this physician particularly alludes, in which his experience is strongly corroborated by my own, viz., the freedom from cardiac and other complications which attended the alkaline treatment. In not a single instance did such complication manifest itself after the patient's admission into the Hospital, and the testimony of the other physicians of the Institution establishes the same fortunate exemption.

I was first led to try the alkaline method by the perusal of Dr. Henry William Fuller's most excellent work on rheumatism. Dr. Fuller, as many are aware, maintains that in acute rheumatism the blood is contaminated by a poison, "which is at once the source and maintainance of the mischief;" and he further asserts, (p. 73,) that this *materies morbi* is an acid, or an acidulous compound, which can be eliminated only by a free exhibition of alkalies and neutral salts. He admits, however, that purgatives, sudorifics, and diuretics, by promoting the various excretions, may assist in accomplishing the object, yet alkalies are essential to prevent the further formation, and to effect the elimination of the morbific element. The remedy preferred by Dr. F., is the potassio tartrate of soda, which is readily decomposed in the stomach, acts quite as energetically, and is much better tolerated than corresponding quantities of the alkaline carbonates. Dr. F. made some experiments to determine the efficacy of impregnating the joints affected with alkaline matter, and though these were not sufficiently extensive to enable him to form a positive opinion, yet, on the whole, they "proved extremely satisfactory." The most serious complication of acute rheumatism, it is needless to add, is heart disease. The frequency with which this occurs, may be inferred from the statistics collected by Dr. Fuller, during the period he held the office of medical registrar at St. George's Hospital, London, viz., from the 1st of January, 1845, to the 1st of May, 1848. Of 379 cases observed by him, embracing all the examples of acute and sub-acute rheumatism admitted, the heart was healthy in 160 instances, in 32 *probably* healthy, while in 187 it was temporarily or permanently deranged to a greater or less extent. These statistics certainly present a most formidable aspect, as some form of disease existed in about half the cases. Now, to preserve the heart from mischief, according to Dr. Fuller, it is necessary not only to take precautions against inflammation, but to maintain the solubility of the fibrin of the blood, and, for this purpose, I am satisfied we must rely on the free administration of alkalies and the neutral salts—the experience of Dr. Fuller, Dr. Griscom, as well as my own, having satisfactorily shown that they surpass all other agents in preventing fibrinous deposits on the valves. Dr. Griscom administered the sup. tart. pot. et soda. in drachm doses, every hour, and ordered an anodyne lotion of carb. potas. and tinct. opii to be applied to the joints affected. Alteratives and evacuants preceded the employment of these remedies, according to the indications presented. The severity of the symptoms was found to diminish as the urine became less acid.

I am aware that Dr. Roderick Macleod, who, at the time of the publication of his work on rheumatism, (1842,) was also physician to St. George's Hospital, has given the results of his experience in the treatment of rheumatism, based on 400 cases attended by him in his public capacity, and that he claims in favor of the blood-letting and purging treatment the average duration attained by Drs. Fuller, Griscom, or myself. For example, of 266 cases, 148 were discharged cured within a month, 110 within a fortnight, and 60 within eight days. Dr. Hope asserted, that under a similar method of treatment, in his practice, the cases were exceptional which were not cured within a week. Dr. Corrigan, with large and repeated doses of opium, cured his cases in nine days. Bouillaud, by copious and repeated abstraction of blood, required from one to two weeks for the same purpose, and yet, if report be true, he lost six out of eighteen patients from cardiac disease.

But I have extended my remarks beyond the limits which I proposed. My only object, in offering a few observations on the alkaline method of treatment, is to bear my humble testimony in its favor, fully believing, as I do, that it is capable of effecting a more speedy and perfect cure than any other plan that has ever been adopted. To those who are skeptical on the subject, I would recommend the study of the facts collected in Dr. Fuller's treatise. In this a comparative view may be taken, which, in my opinion, must satisfy every unprejudiced mind. It will there be found, that the boasted success of the authors whom we have mentioned, admits of considerable qualification, and is in reality less than has been asserted.—*Western Lancet.*

